# High incidence of periprosthetic joint infection with propionibacterium acnes after the use of a stemless shoulder prosthesis with metaphyseal screw fixation - a retrospective cohort study of 241 patients propionibacter infections after eclipse TSA

**DOI:** 10.1186/s12891-017-1555-8

**Published:** 2017-05-19

**Authors:** Lisa Johansson, Nils P Hailer, Hans Rahme

**Affiliations:** Institute of Surgical Sciences, Department of Orthopaedics, Uppsala University Hospital, Uppsala, Sweden

**Keywords:** *Propionibacterium acnes*, Shoulder arthroplasty, Periprosthetic infection, Biofilm formation, Orthopedic implant, TSA, TSR

## Abstract

**Background:**

A stemless shoulder prosthesis with humeral metaphyseal screw fixation was introduced in order to save bone-stock and to facilitate reconstruction of biomechanics (Eclipse®). The aim of this study was to analyze whether the risk of infection is different with this implant compared to conventional shoulder prosthesis.

**Methods:**

Two hundred and forty-one patients (54.8% females) were operated with a shoulder arthroplasty and followed for median 2.0 (0.1–5.7) years. One hundred and two (42.3%) had received an Eclipse® prosthesis, the remaining patients were operated with other implants. There was an overrepresentation of males in the Eclipse® group (63.7% males) when compared with the control group (31.7% males).

**Results:**

In the Eclipse® group 10 (9.8%) patients developed a periprosthetic joint infection, as opposed to 1 (0.7%) in the control group. The most common bacteria was *Propionibacterium acnes*. Unadjusted infection-free survival after 4 years was 88.8% (CI 82.5–95.7) for Eclipse® patients and 95.7% (CI 87.7–100.0) for controls (*p* = 0.002). After adjustment for age, gender, diagnosis, and type of shoulder prosthesis (total or hemi), the risk ratio for revision due to infection was 4.3 (CI 0.5–39.1) for patients with the Eclipse® prosthesis.

**Conclusions:**

Deep infections seem to be more common after the use of the metaphyseally fixed Eclipse® prosthesis than after conventional shoulder implants, but a predominance of male gender and younger age in the Eclipse group may have biased our findings. Future studies on larger cohorts and in vitro investigations on bacterial adherence and biofilm formation are needed.

**Trial registration:**

The study was conducted in accordance with the Helsinki Declaration. The local ethics board (Regionala Etikprövningsnämnden Stockholm) approved the study (Approval number 2015/1590–31, date of issue 2015-10-14). Retrospectively registered.

## Background

The Eclipse® prosthesis is a stemless shoulder prosthesis enabling anatomic reconstruction of the center of rotation independently from the head and shaft relationship. The osteotomy through the anatomical neck facilitates access to the glenoid when compared with a resurfacing shoulder prosthesis. The Eclipse® device consists of a cage screw, a trunnion manufactured of titanium alloy (ISO 5832–3), and a humeral head manufactured of cobalt- chromium alloy (ISO 5832–12). At our large-volume shoulder arthroplasty unit the metaphyseally anchored Eclipse® prosthesis was introduced in 2008. The observation that a larger-than expected number of patients treated with this prosthesis returned with signs of periprosthetic infection and required subsequent revision surgery triggered a suspicion that this specific device might be associated with a higher risk of infection.

Periprosthetic infection is a serious but rare complication following shoulder arthroplasty [23]. Today *Propionibacterium acnes* (*P. acnes*) has emerged as the most common pathogen in implant-associated shoulder infections, and it has even been suggested that *P. acnes* could be causative in the development of shoulder osteoarthritis [16]. *P. acnes* is a gram-positive anaerobic microorganism that colonizes the skin above the shoulder girdle more frequently than the skin surrounding the hip or knee, and males have a higher *P. acnes* burden than females [13, 22].

The surface of an implant is crucial for the formation of bacterial biofilms and textured mammary implants develop a higher load of bacterial biofilm than smooth implants [10]. Biofilm formation on different titanium alloys and polymethylmetacrylate bone cement has been found with different strains of *P. acnes* in vitro [8]. Antibacterial coatings and surface modifications on orthopedic implants to render the implant surface antibacterial properties have been studied extensively in vitro [12]. To our knowledge, no study addresses the question whether certain types of shoulder implants are more prone to colonization by *P. acnes* than others.

The purpose of this retrospective study was to evaluate the infection rate after shoulder replacement with the Eclipse® prosthesis and to compare it with the infection rate following other shoulder replacements at our department.

## Methods

### Study design and data collection

**Fig. 1:**
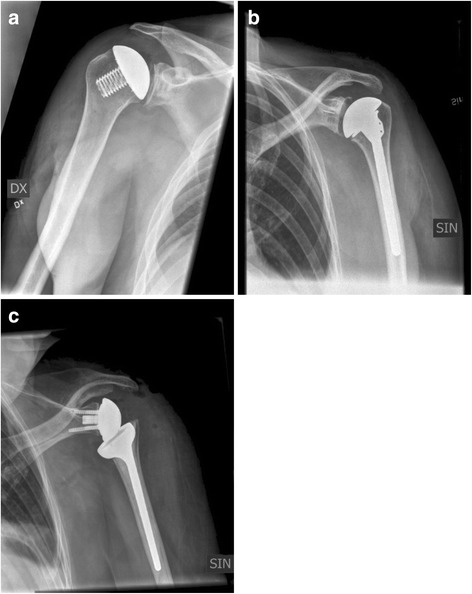
Showing the different types of prosthesis included in the study. Figures showing (**a**) Eclipse, (**b**) Delta Xtend, (**c**) Bigliani

This study is a retrospective cohort study. The study was conducted in accordance with the Helsinki Declaration. The local ethics board (Regionala Etikprövningsnämnden Stockholm) approved the study (Approval number 2015/1590-31, date of issue 2015-10-14). Since we hypothesized that the infection rate might be higher in the group of patients operated with an Eclipse® prosthesis we divided all patients operated with a shoulder arthroplasty at our unit during the study period into those that had received this specific implant and a control group consisting of all other patients operated with a different type of shoulder prosthesis during the same period (see Fig. 1). All arthroplasties were performed by two experienced shoulder surgeons in the same operating rooms and with the same staff. Both surgeons are experienced regarding all types of shoulder prosthesis.

Collected data included the date of index surgery, age, gender, preoperative diagnosis, perioperative antibiotics, implant type, and incidence of postoperative infection. In patients with a postoperative infection information on the time from the date of index surgery to diagnosis of infection, counts of white blood cells (WBC), erythrocyte sedimentation rate (ESR), concentration of C-reactive protein (CRP), frontal and axial view radiographs, date of revision, type of revision, type of bacteria found upon microbiological investigation, and the result after revision were collected.

In order to ensure that patients had not been revised elsewhere we cross-validated our database with the Swedish Shoulder Arthroplasty Register where all revision procedures are registered on a national basis. We identified one patient that had been subject to revision surgery of the index joint at an external unit.

### Surgical procedures

At the time of index surgery all patients received perioperative intravenous antibiotics prior to incision. The antibiotic regimen was cloxacillin 2 g + 1 g + 1 g + 1 g, or, in cases of penicillin allergy, clindamycin 600 mg × 3 during 24 hours. Skin disinfection was performed with chlorhexidine (5 mg/ml, Fresenius Kabi). All patients underwent a general anesthesia in combination with interscalene brachial plexus block. A deltopectoral approach was used in all cases, the subscapularis tendon was incised 10 mm medial to the lesser tuberosity and the shoulder dislocated. An osteotomy was carried out through the anatomical neck in 20 to 30° of retroversion. Subsequently, in cases receiving a total shoulder arthroplasty, the glenoid was prepared and an all polyethylene glenoid component was inserted using Palacos R + G (Heraeus Nordic). In cases receiving the Eclipse® prosthesis the trunion was secured with a cage screw and a humeral head was impacted onto the trunion. After reduction and routine assessment of joint stability and range of motion the subscapular tendon was sutured with Ortocord (Johnsson & Johnsson), and the wound was closed layer by layer with Vicryl and an intradermal Monocryl suture.

### Microbiology

Eight of 10 patients with suspected periprosthetic infection in the Eclipse® group underwent a diagnostic shoulder arthroscopy before revision surgery was performed. During the arthroscopy several tissue samples (4–9) were collected using sterile procedures with separate biopsy instruments. In the remaining 2 of the 10 patients with suspected infection in the Eclipse® group, no diagnostic arthroscopy was performed, but tissue samples were obtained during revision surgery. The patient in the control group with a periprosthetic infection developed a fistula and cultures were obtained from this fistula. All tissue samples were sent to the Department of Clinical Microbiology, Uppsala University Hospital, for microbiological analysis. The tissue samples were divided into several cultures; chocolate blood agar (CBA) for aerobic incubation, fastidious anaerobic agar (FAA) for anaerobic incubation, and two serum broths. CBA and FAA were incubated at 35 °C (±1 °C), FAA in the presence of 10% carbon dioxide, and analyzed after two to four days in vitro. The broths were enriched and analyzed after 10 days. All samples were investigated by laboratory personnel on a daily basis.

### Characteristics of the study population

The study population consisted of a consecutive series of 241 patients who were operated from 2008-09-24 to 2012-10-24 at our unit. None of the patients was operated bilaterally, 132 (54.8%) were females, and the mean age at surgery was 63.9 (SD 11.1) years. One hundred and sixty-five (68.5%) patients had primary glenohumeral osteoarthritis as the underlying diagnosis, and 102 (42.3%) received the Eclipse® prosthesis, whereas the remaining patients received one of five different other implants (see Table 1).

**Table 1:** Summary of characteristics of the study population

Description of the study population	Control No.	%	Eclipse No.	%
Gender				
Male	44	31.7	65	63.7
Female	95	68.3	37	36.3
Age				
[0,65)	47	34.8	67	65.7
[65,90)	88	65.2	35	34.3
Diagnosis				
prim. OA	73	52.5	92	90.2
Cuff disease	34	24.5	0	0
ON	1	0.7	0	0
RA	11	7.9	0	0
sec. OA	20	14.4	10	9.8
Implant				
Eclipse	0	0	102	100
Bigliani	32	23	0	0
CTA	7	5	0	0
Delta	47	33.8	0	0
Epoca	4	2.9	0	0
Univers	49	35.3	0	0
Total or hemi				
Hemi	24	17.3	10	9.8
Total	115	82.7	92	90.2

Indication for the Eclipse® prosthesis was primary or secondary arthritis and good bone quality. Total shoulder arthroplasties were performed in 207 (85.9%) patients, the remaining patients were treated with hemiarthroplasties. The mean follow-up time was 2.1 (range 0.1 to 5.7) years.

In order to investigate our primary hypothesis, we divided the study population into a group of patients who had received the Eclipse® prosthesis (“Eclipse”) and a group of patients who had received a different implant (“Control”). Males were more common in the Eclipse group (63.7%) than among control patients (31.7%, *p* < 0.001). The proportion of patients younger than 65 years was larger in the Eclipse group (65.7%) than among controls (34.8%, *p* < 0.001). There were more hemiarthroplasties in the control group than in the Eclipse group, but this difference failed to reach the level of statistical significance (*p* = 0.145). Details on the distribution of gender, age, diagnoses, and implant types over the two investigated groups are given in Table 1.

### Statistics

Database management was performed using Microsoft Excel 2007–2010 ® software. Statistical analysis was performed using R software version 3.1.3 together with the “rms” and “Gmisc” package [24]. Continuous data were described using means, medians, standard deviations and ranges. 95% confidence intervals (CI) were used to describe estimation uncertainty. Categorical data were summarized in cross-tables and the Chi-square test was used in order to investigate differences between groups. The Kaplan-Meier method was used to calculate cumulative unadjusted component survival with the endpoint appearance of infection, and Mantel-Haenszel’s log-rank test was applied in order to assess whether survival differed between groups. Cox multivariable regression models were fitted in order to calculate hazard ratios for the risk of experiencing the primary endpoint, adjusted for relevant confounders. Scaled Schönfeld-residuals were plotted and calculated in order to verify that the assumption of proportional hazards was fulfilled. *p*-values < 0.05 were considered statistically significant. A post-hoc power analysis indicated that our study with 102 patients in the Eclipse group and 139 patients in the control group had a statistical power of 83% to detect a 9-fold increase in the infection frequency in the Eclipse group with a type-I error rate (alpha) of 0.05. More subtle differences between infection frequencies in the two groups would accordingly have required much larger cohorts.

## Results

Patients with shoulder pain at rest and without probable other causes were suspected to have a periprosthetic joint infection. Routinely, these patients were examined with ultrasound to ensure that no pathology such as rotator cuff tear was the reason behind the complaints. Infection was defined as bacterial growth in more than 2 out of 5 cultures. At follow-up, a total of 11 (4.6%) patients suffered from a periprosthetic joint infection, and 10 of these patients had received the Eclipse® implant. The most common bacteria, *P. acnes*, were isolated from 8 patients, all of them in the Eclipse® group. Malalignment was detected in 1 patient and prosthetic loosening in 1. Several patients were discovered to have a loose glenoid component at revision surgery. Out of 10 infected patients, 8 underwent revision surgery at our unit, 4 of these were 1-staged and 4 were 2-staged with a spacer. Of the 2 patients who did not undergo revision surgery at our unit one was treated with antibiotics only and the other was revised elsewhere. All patients, including those that were treated elsewhere, experienced a satisfying outcome. There were no reinfections after revision surgery.

Further details on the infected patients are given in Table 2.

**Table 2:** Details on patients with periprosthetic infection

Description of infected patients	Control	Eclipse
Gender		
Male	0	10
Female	1	0
Diagnosis		
prim. OA	1	8
sec. OA	0	2
Implant		
Eclipse	0	10
Univers	1	0
Total or hemi		
Hemi	0	4
Total	1	6
Bacteria		
Propioni	0	6
CoNS	1	2
Prop. + CoNS	0	2

**Fig. 2:**
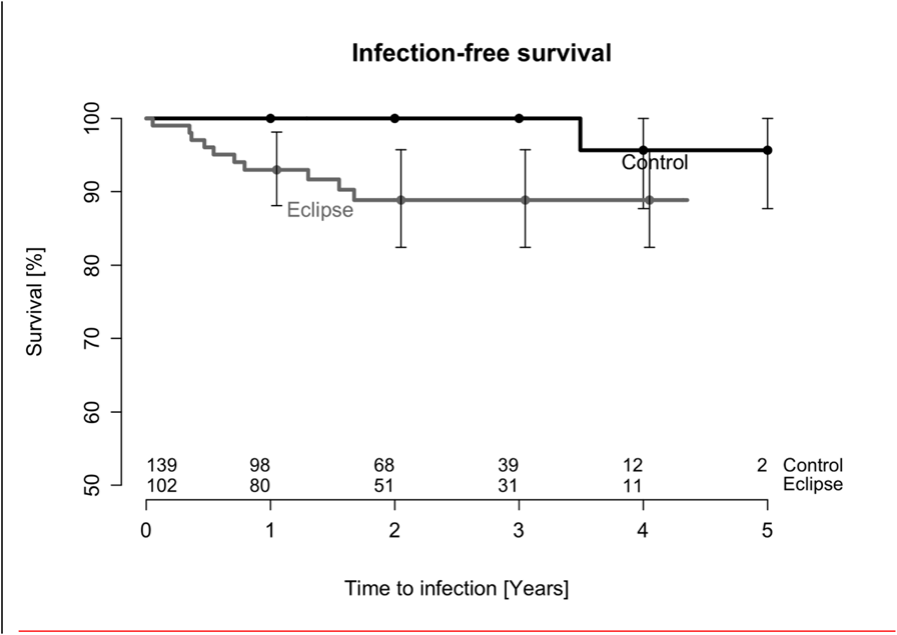
Unadjusted infection-free survival

Kaplan-Meier analysis with the endpoint appearance of infection was performed in order to calculate implant survival at different time points. We found that unadjusted infection-free survival after 4 years was 88.8% (CI 82.5-95.7) for the Eclipse® group and 95.7% (CI 87.7-100.0) for controls, a difference that was statistically significant (*p* = 0.002, see Fig. 2).

Since gender and age were asymmetrically distributed between the Eclipse® and the control group we performed a multivariable Cox regression analysis in order to adjust for these and other potential confounders. The crude risk of revision due to infection was 12.3-fold (CI 1.6-96.3) for Eclipse® patients when compared with controls. After adjustment for age, gender, and the type of arthroplasty (hemiarthroplasty or total arthroplasty) the risk ratio for Eclipse® patients was 4.3 (CI 0.5-39.1). The risk estimates were similar irrespective of whether age was treated as a categorical or a continuous variable, see Table 3.

**Table 3:** Adjusted risk of revision due to infection

Risk of infection	Crude HR	2.5% CI	97.5% CI	Adj. HR	2.5% CI	97.5% CI
Implant						
Eclipse	12.3	1.6	96.3	4.3	0.5	39.1
Gender						
Female	0.1	0	0.6	0.2	0	1.8
Hemi or Total						
Total	0.3	0.1	1.1	0.4	0.1	1.6
Age						
[65,90)	0.1	0	0.8	0.3	0	2.4

## Discussion

### Main findings

In this retrospective study we found that patients who received an Eclipse® prosthesis had an increased risk of infection compared to a control group. The most common bacteria were *P.* acnes that were isolated in 8 of 11 patients, all of them in the Eclipse® group. Our total infection rate was at the upper limit of what is reported from large shoulder arthroplasty series [14, 23], and the accumulation of these infected cases in a group of patients treated with a specific type of implant is a matter of concern. We here discuss whether this observation may be related to the implant or to patient-specific factors.

### Periprosthetic joint infection after shoulder arthroplasty

In early postoperative prosthesis infections occurring within three months after index surgery the causative agents are mostly *S. aureus*. In delayed infections that mostly appear between three months and two years after index surgery the causative agents are often less virulent bacteria from the normal skin flora, such as *S. epidermidis* [17], or, after shoulder arthroplasty, *P. acnes* [23]. *P. acnes* was isolated in deep cultures obtained from 42% of shoulders operated with an arthroplasty for osteoarthritis [16], leading the authors to speculate that this microorganism might be causative for the development of osteoarthritis. This hypothesis was contested by Maccioni et al. who found a low rate of *P. acnes* in osteoarthritic shoulders undergoing total shoulder replacement [18], a discrepancy that is possibly due to differences in the specimen collection technique.

Whatever the cause-effect-relationship between *P. acnes* and glenohumeral osteoarthritis, there is little doubt that this microorganism is found in a substantial proportion of cultures obtained during either primary or revision surgery of the shoulder. *P. acnes* were found in 24% of shoulders undergoing revision arthroscopy for various reasons [9], and the same organism were isolated from deep tissue culture specimens obtained during open shoulder surgery [20], even despite timely antibiotic administration [19]. Male gender and younger age seem to predispose towards *P. acnes* colonization [13].

Prevention of surgical site contamination with *P. acnes* seems difficult since this microorganism persists in the dermis even after surgical site preparation with chlorhexidine [13, 15]. The efficacy of different antibiotics against *P. acnes* is controversially discussed, but a survey of *P. acnes* isolates from shoulders over a one-year period indicates that penicillin G as well as cephalosporins display acceptable minimal inhibitory concentrations, a finding that is consistent with earlier reports on the susceptibility of *P. acnes* to different antibiotics [4, 26]. It is equally controversial whether standard incubation times suffice for the detection of *P. acnes*, or whether prolonged culture periods are required [2, 25].

There are three different mechanisms behind the development of periprosthetic joint infection: direct intraoperative contamination, haematogenous dissemination, and reactivation of latent infection [5]. An interdependent relationship exists between bacterial virulence, a patient’s immune status, and the local wound environment [11]. A small bacterial inoculum during surgery must not necessarily lead to periprosthetic joint infection [3, 11], and perioperative intravenous antibiotic prophylaxis aims at eradicating contaminating bacteria from the situs. The absence of systemic antibiotic prophylaxis is a major risk factor for the development of infection after primary hip arthroplasties [6, 7], and – although we are not aware that similar studies exist with regard to shoulder arthroplasty – the same line of reasoning seem probable even in that joint. Thus, systemic antibiotic prophylaxis is routinely administered prior to shoulder arthroplasty, and in agreement with national routines we used cloxacillin at an adequate dose.

Our observation of a high proportion of *P. acnes* in infected shoulder arthroplasties is in line with previous reports. Furthermore, the susceptibility of younger males – i.e., the type of patient that dominated the Eclipse® group – to colonization and subsequent infection may have contributed to the observation of an increased number of infections in the Eclipse® group. Our awareness of periprosthetic infections after shoulder arthroplasty made us follow a quite stringent diagnostic algorithm based on arthroscopy and acquisition of tissue cultures, but this is in agreement with literature describing infection with *P. acnes* as a reason for otherwise unexplained pain after shoulder surgery [9].

### Implant-related factors underlying infection

Apart from patient-related factors mentioned above another possible explanation for our observation of a higher-than anticipated rate of infections in patients operated with the Eclipse® device may also be found in the material and structure of this implant. The trunnion and cage screw of this implant are manufactured from a titanium alloy that has high biocompatibility and that is considered ostoeconductive. These desired effects are on the one hand related to enhanced bony ingrowth, on the other hand biocompatibility can also be associated with an increased potential of bacterial colonization and biofilm formation [27]. In vitro investigations show that *P. acnes* can adhere to and form biofilm on various titanium alloys, furthermore, that both bacterial adherence and the capacity for biofilm formation are dependent on surface roughness of the material in various bacterial strains, including *P. acnes* [1, 8]. Copper oxide coatings of titanium alloys seem to reduce the density of planktonic *p. acnes* and also inhibit the formation of biofilm, but such coatings are still at an experimental stage [21].

Having said that, the Eclipse® prosthesis is of course not the only implant made of titanium alloys, thus, the susceptibility of this specific implant to infection with *P. acnes* cannot solely be explained by the type of alloy it is manufactured from. However, the metaphyseal cage screw entirely made of a roughly textured, porous titanium alloy is unique to this type of implant, and – given the findings derived from the in vitro studies cited above – surface roughness and alloy composition exert an important influence on bacterial colonization and biofilm formation. It is tempting to speculate that the excellent biocompatibility of this crucial component of the investigated implant may simultaneously cause it to be more susceptible to colonization with *P. acnes*. However, further in vitro investigations using this and other types of shoulder implants, including other stemless shoulder prosthesis, are necessary before firm conclusions on this topic can be drawn. Further studies would also specify whether the material or the helical screw might influence the risk of bacterial colonization.

### Strengths and weaknesses of the current study

Ours is a retrospective cohort study with all typical weaknesses inherent to such a design. Retrospective data collection from medical charts is always less reliable than prospective registration. On the other hand, by cross-validating our findings with the Swedish Shoulder Arthroplasty Register we know that one patient in our cohort was revised at a different unit, thus, we feel quite confident in the completeness of our follow-up as far as the endpoint revision surgery is concerned. There could be cases with latent infection that have not shown up with complaints at our unit, but there is no reason to assume that the resulting failure to detect infections should be unequally distributed between the two groups of patients, i.e., “Eclipse” and “Control” patients. Another advantage of our study is that we follow an entire cohort of patients operated upon during a defined period of time, with no exclusion of any patient for whatever reasons. Our unadjusted survival analysis with the endpoint “occurrence of infection” indicated a statistically significant difference between the two groups of patients. Since the gender and age proportions differed considerably between “Eclipse” and “Control” patients we performed an adjusted regression analysis, and the relative risk of infection was still 4-fold increased in “Eclipse” compared with “Control” patients. Due to the low number of events (11 infections in the entire cohort of 241 patients) confidence intervals around these risk estimates were wide, and the adjusted risk of infection was no longer statistically significantly different between the two groups. However, the occurrence of 10 infections in 102 “Eclipse” patients compared with 1 infection in the remaining 139 ”Control” cases is a finding that raises suspicions. There was a difference in gender and age proportions between the two groups. There were more men in ”Eclipse” compared to the ”Control” group, and patients in the ”Eclipse” group were younger at the time of index surgery. Therefore, the ”Eclipse” group should rather had fewer comorbidities, potentially reducing the risk of periprosthetic infection compared to the”Control” group. On the other hand, males are more prone to developing *P. acnes* infections, and the predominance of males in the ”Eclipse” group may make this group more susceptible to periprosthetic joint infection. Taken together, the lack of reliable data on medical comorbidities is a weakness of our study.

## Conclusions

Deep infections with *P.* acnes seem to be more common after the use of the metaphyseally fixed Eclipse® prosthesis than after conventional shoulder implants. This finding was unexpected, and to our knowledge this is the first report describing this phenomenon. Younger males are more susceptible to periprosthetic joint infection of the shoulder with *P. acnes*, and the predominance of male gender and younger age in the Eclipse® group may have biased our findings to the disadvantage of the Eclipse® prosthesis since. It is possible that the described prosthesis is more prone to bacterial colonization, but in vitro investigations on the ability of different bacteria to colonize this implant – compared with other stemless implants – should be undertaken. Until further evidence has been presented we refrain using this specific implant.

## Abbreviations

CBA: Chocolate blood agar
CI: Confidence interval
CRP: C-reactive protein
ESR: Erythrocyte sedimentation rate
FAA: Fastidious anaerobic agar
*P. acnes*: *Propionibacterium acnes*
SD: Standard deviation
TSA: Total shoulder arthroplasty
TSR: Total shoulder replacement
WBC: White blood cells
