# Erratum to: High incidence of periprosthetic joint infection with propionibacterium acnes after the use of a stemless shoulder prosthesis with metaphyseal screw fixation - a retrospective cohort study of 241 patients propionibacter infections after eclipse TSA

**DOI:** 10.1186/s12891-017-1610-5

**Published:** 2017-06-08

**Authors:** Lisa Johansson, Nils P. Hailer, Hans Rahme

**Affiliations:** Institute of Surgical Sciences, Department of Orthopaedics, Uppsala University Hospital, Uppsala, Sweden

## Erratum

After the publication of the article [1] is was brought to our attention that the title was incorrectly given as “High incidence of periprosthetic joint infection with propionibacterium acnes after the use of a stemless shoulder prosthesis with metaphyseal screw fixation - a retrospective cohort study of 241 patients propionibacter infections after eclipse TSA”. The correct title is “High incidence of periprosthetic joint infection with propionibacterium acnes after the use of a stemless shoulder prosthesis with metaphyseal screw fixation - a retrospective cohort study of 241 patients“.
